# Using metabarcoding to reveal and quantify plant-pollinator interactions

**DOI:** 10.1038/srep27282

**Published:** 2016-06-03

**Authors:** André Pornon, Nathalie Escaravage, Monique Burrus, Hélène Holota, Aurélie Khimoun, Jérome Mariette, Charlène Pellizzari, Amaia Iribar, Roselyne Etienne, Pierre Taberlet, Marie Vidal, Peter Winterton, Lucie Zinger, Christophe Andalo

**Affiliations:** 1 Laboratoire Evolution and Diversité Biologique EDB, Université Toulouse III Paul Sabatier, F-31062 Toulouse, France; 2 CNRS, EDB, UMR 5174, F-31062 Toulouse, France; 3 Laboratoire Biogeosciences, Université de Bourgogne 6 bld Gabriel, F-21000 Dijon, France; 4 Plate-forme Bio-informatique Genotoul, Mathématiques et Informatique Appliqués INRA, UR875 Toulouse, F-31320 Castanet-Tolosan, France; 5 Laboratoire d’Ecologie Alpine, CNRS UMR 5553, Université Joseph Fourier, BP 43, F-38041 Grenoble, France; 6 GeT-PlaGe, Genotoul, INRA UAR1209, F-31320 Castanet-Tolosan, France; 7 Département de Langues et Gestion, Université Paul Sabatier, F-31062 Toulouse, France

## Abstract

Given the ongoing decline of both pollinators and plants, it is crucial to implement effective methods to describe complex pollination networks across time and space in a comprehensive and high-throughput way. Here we tested if metabarcoding may circumvent the limits of conventional methodologies in detecting and quantifying plant-pollinator interactions. Metabarcoding experiments on pollen DNA mixtures described a positive relationship between the amounts of DNA from focal species and the number of *trnL* and ITS1 sequences yielded. The study of pollen loads of insects captured in plant communities revealed that as compared to the observation of visits, metabarcoding revealed 2.5 times more plant species involved in plant-pollinator interactions. We further observed a tight positive relationship between the pollen-carrying capacities of insect taxa and the number of *trnL* and ITS1 sequences. The number of visits received per plant species also positively correlated to the number of their ITS1 and *trnL* sequences in insect pollen loads. By revealing interactions hard to observe otherwise, metabarcoding significantly enlarges the spatiotemporal observation window of pollination interactions. By providing new qualitative and quantitative information, metabarcoding holds great promise for investigating diverse facets of interactions and will provide a new perception of pollination networks as a whole.

The ongoing global decline of both pollinators^1^,^2^ and plants^3^ emphasizes the need for a better understanding of the structural and functional characteristics of pollination networks worldwide^4^. Pollination networks also constitute powerful models to investigate the role of ecological interactions in the formation and the maintenance of biodiversity^5^,^6^. However, a comprehensive description of these networks across time, space and in a context of global changes represents a formidable challenge as many plants and animals are involved, with numerous possible links between them.

Much of our current knowledge about plant-pollinator interactions relies on studies of simplified pollination systems involving a single or a few focal plant species with their pollinators only. These systems might, however, lead to unrealistic inferences, as they are not representative of the complexity of most pollination networks. Moreover, the conventional approaches used to describe plant-pollinator interactions, based on pollinator visit records, has several severe limits. First, it commonly assumes that visitors interact positively with plants. In reality, a visiting animal may interact negatively with a plant by robbing nectar or pollen without providing pollination services^7^. It can also deposit heterospecific or incompatible conspecific pollen onto the stigma^8^. The transfer of inappropriate pollen may lead to indirect negative interactions between co-flowering plants^8^ and hamper the advantage they may derive from the co-attraction of pollinators^9^. Second, visitation records are very sensitive to sampling effort, *i.e.* the longer the observations are, the more interactions will be observed. Third, they suffer from taxonomic impediments due to the difficulties in identifying many insect species in the field. Finally, they do not reveal particular behaviors of individuals or colonies. Accordingly, recent studies have shown that the flower-visitor network may only faintly describe real interaction networks^10^,^11^,^12^,^13^.

Because both the type (positive, neutral, negative) and the force of interactions between plant and visitor species can leave a footprint on insect and stigma pollen loads, quantifying and identifying plant species that make up pollen loads will provide essential information on plant-pollinator and plant-plant interactions. Unfortunately, pollen identification is time-consuming, requires expertise and even so is often limited to the genus or the family levels^14^. Together, these methodological flaws may hamper the reconstruction of realistic pollination networks. This may seriously limit our understanding of the ecological and evolutionary processes shaping these networks^5^,^6^ and predictions about how they would respond to environmental perturbations^15^,^16^.

Here, we tested metabarcoding to determine whether it has the potential to circumvent these limits. Briefly, the study aims at identifying taxa from the DNA contained in environmental samples through PCR amplification and next-generation sequencing of DNA contained in the samples^17^. Metabarcoding is now widely used to characterize the taxonomic composition and richness of plant or invertebrate communities from soil or sediment samples^18^,^19^,^20^ and could also be applied to insect and stigma pollen loads as well. However, to be relevant for unravelling plant-pollinator networks, metabarcoding must provide reliable identification and optimally, accurate quantification of plant species in pollen loads on visitors and stigmas. DNA extraction and sequencing of a very small number of pollen grains is feasible^21^ and the pollen carried by insects can be used to reveal pollination interactions by DNA barcoding^14^,^22^,^23^,^24^. However, the suitability of metabarcoding to quantify species abundance in environmental samples has been repeatedly questioned for different systems^19^,^25^ and more recently for pollen samples^24^.

We evaluated the suitability of metabarcoding at revealing and quantifying plant-pollinator interactions by using two plant specific molecular markers, the chloroplastic *trnL* gene and the nuclear ITS1 intron and MiSeq Illumina sequencing. First, from artificial pollen DNA mixtures of three different plant species, we investigated (i) if, within a species, the number of pollen grains and the number of ITS1 and *trnL* sequences yielded were proportionally related. Pollinators commonly carry a mixture of pollen grains from different species and in various and often unequal abundances. Therefore we had to check (ii) if the relationship between the number of pollen grains and the number of sequences for a focal species held true regardless of the relative abundances and identities of the other plant species (hereafter called neighboring species) in the pollen mixtures. Second, we captured insects in subalpine communities dominated by *Rhododendron ferrugineum* in the French Central Pyrenees (Camurac southern France). We identified plant species in insect pollen loads and examined (i) if the amounts of ITS1 and *trnL* sequences obtained from insect pollen loads are related to the pollen-carrying capacity of insects and (ii) if the amounts of ITS1 and *trnL* sequences obtained from pollen loads were related to insect visit frequency.

## Results

### Artificial pollen DNA mixtures

**Figure 1:**
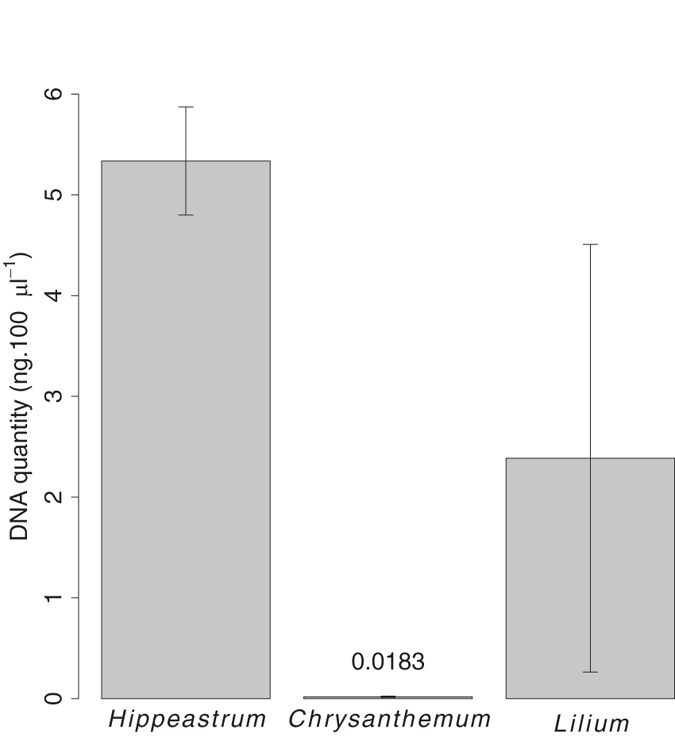
Total DNA yields (ng 100 μl^−1^, mean ± SD, n = 5) from 10,000 pollen grains of *Lilium sp. Hippeastrum sp.* and *Chrysanthemum sp*.

We examined the relationships between the number of pollen grains and the number of ITS1 and *trnL* sequences obtained for three plant species (*Chrysanthemum sp., Hippeastrum sp.,* and *Lilium sp.*) either alone or in mixture. The extraction of total DNA from the same number of pollen grains (10,000 here) gave very different DNA yields among the three plant species (Fig. 1). Before sequencing, equimolar pools were constructed from PCR products containing different amounts of DNA. For this reason the number of sequences obtained for each sample was weighted for the proportion of PCR product added to the pool relative to the total amount of PCR products.

**Figure 2:**
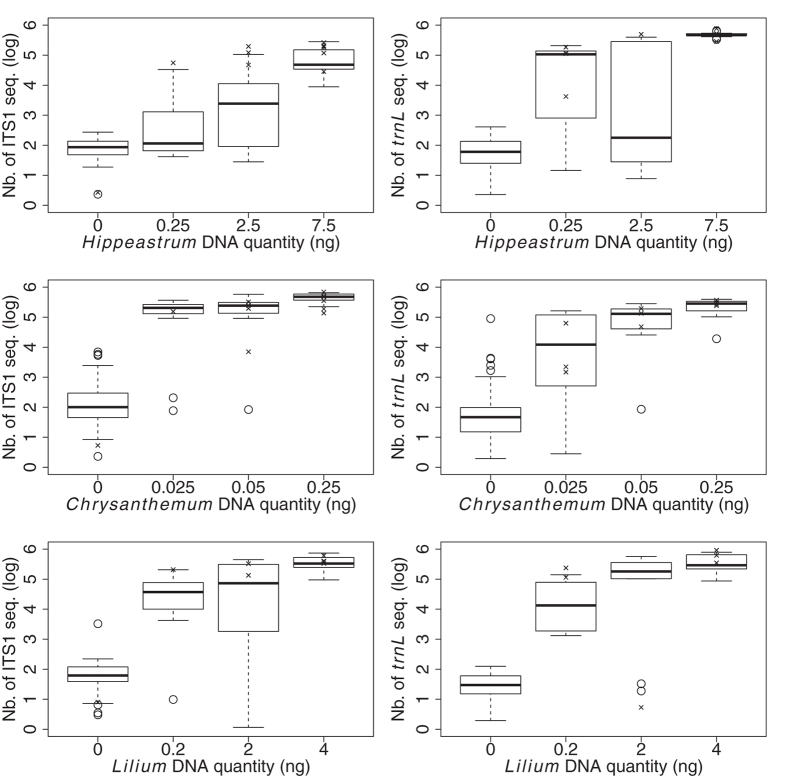
Boxplot showing the number of ITS1 or *trnL* sequences obtained from varied amounts of pollen DNA from *Lilium sp.*, *Hippeastrum sp.* and *Chrysanthemum sp.* in artificial mixtures. The crosses indicate the number of sequences obtained when the focal species was alone.

On average, *Chrysanthemum sp.* (CHR) produced 290 and 130 times less DNA than *Hippeastrum sp.* (HIP) and *Lilium sp.* (LIL) respectively. LIL showed high intra-specific variation in DNA yield (coefficient of variation, CV: 89%). The variation was lower for CHR (CV: 36%) and even more so for HIP (CV: 10%). Still, lower DNA amounts (CHR: 0.025 ng; HIP: 0.25 & LIL: 0.2 ng) corresponding to a few pollen grains (125, 5 & 4 pollen grains respectively) gave frequently more than 1000 sequences for both ITS1 *and trnL* markers (Fig. 2).

We found a positive relationship between the amount of template pollen DNA of each species and the number of corresponding sequences generated, whatever the marker used or mixture diversity (Fig. 2, Table 1 and Table S1). However, variability among replicates was high and we were unable to retrieve sequences for some of them, probably because the PCR amplification did not work. Furthermore, the identity of the added species in the mixture impacted the number of sequences harvested and reduced the strength of the relationship between the amount of DNA and the number of sequences of the focal species. The identity effect was especially strong when CHR was the added species in the mixture. The DNA amount of the added species did not itself affect the relationship but acted significantly in interaction with the identity of the added species (Table 1).

For the three species, the greatest variability arose (Fig. 2) principally for low DNA amounts (Fig. 2) corresponding to very few (HIP, LIL) or a few pollen grains (CHR; Table 2). With greater amounts of DNA, still corresponding to a relatively low number of HIP and LIL pollen grains (150 and 80 respectively), the variability in harvested plant sequences and the effect of added species in the mixtures were both much more limited. For instance, we detected noticeable and more variable amounts of CHR sequences in pollen mixtures where that species was not introduced (Fig. 2).

### Analyses of insect pollen loads

We investigated plant species composition and abundance in pollen loads obtained from 402 insects captured in four *Rhododendron ferrugineum* communities. We assessed the relationship of the amounts of ITS1 and *trnL* sequences with insect pollen-carrying capacity, known from other studies (see Materials and Methods), as well as with the insect visit frequency recorded visually in the field during the capture sessions. We expected that the number of ITS1 and *trnL* sequences would reflect the number of pollen grains (either for all community plant species or for the target species *R. ferrugineum*) in insect pollen loads and/or the number of visits (all visitors pooled) received by a plant species. The sequences were identified using both the EMBL and our local reference database (see methods), the latter comprising the plant species occurring at the study site.

Among the 402 insects studied (Table S2), 76 (19%) and 53 (13%) produced neither *trnL* nor ITS1 plant sequences respectively, and 13 individuals (≈3%) did not produce any sequences at all. Before sequencing, equimolar pools were constructed from PCR products containing different amounts of DNA. For this reason the number of sequences obtained for each sample was weighted for the proportion of PCR product added to the pool relative to the total amount of PCR products. After the weighting, the remaining 389 samples rendered, a total of 76,194,449 ITS1 sequences for which we were able to assign 99.65% to a plant taxon with 66.95%, 18.70%, 13.95%, 0.04% being assigned at the species, genus, family and class plant taxonomic levels respectively. Similarly, of the 37,886,420 plant sequences obtained with the *trnL* marker, 75.85%, 5.36%, 13.25%, 0.21%, and 4.70% were assigned to the species, genus, family, class and order taxonomic levels respectively (99.38% sequences were assigned to a plant taxa in total).

The sequences corresponded to 74 entomophilous plant taxa (63 species, 11 genera) and 17 anemophilous/amphiphilous plant taxa (7 species, 10 genera; Table 3). Among the 63 entomophilous plant species identified, 23 were observed only in the surrounding landscape and not in the studied communities. Coniferous and grass taxa were the most prevalent anemogamous species in the DNA sequences, which was in agreement with their abundance in the communities studied or surrounding landscape.

Thus, as compared to the observation of visits (25 entomophilous species visited, Table 3) metabarcoding revealed 2.5 times more plant species involved in plant-pollinator interactions. Only two species (*Melampyrum sylvaticum*, *Trifolium pratense*), which received only one visit each, were not detected with metabarcoding. Moreover, 19 species represented by few individuals and visibly not visited during capture sessions were not detected in sequence data either (Table 3).

**Figure 3:**
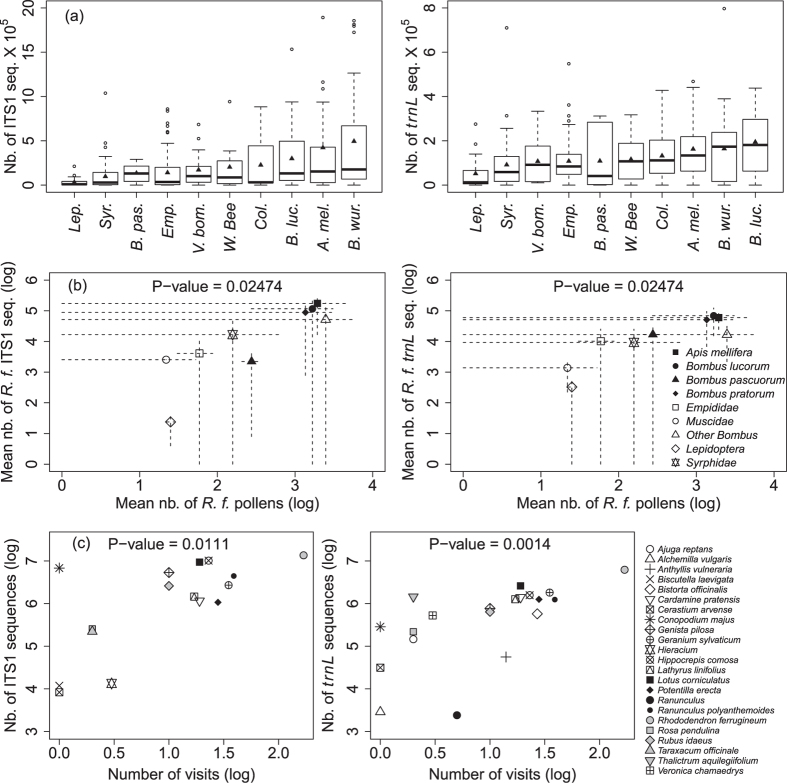
Quantification of plant-pollinator interactions in the *Rhododendron ferrugineum* communities (**a**) Boxplot of the number of total ITS1 and *trnL* sequences obtained from pollen loads of the main insect taxa. Triangles represent the mean values. One *A. mellifera* individual with more than 4,000,000 ITS1 sequences was not included in the figure, (**b**) Relationship between the number of pollen grains and the number of ITS1 and *trnL* sequences of *R. ferrugineum* across insect taxa (dashed lines represent standard deviations), (**c**) Relationship between the number of visits received by each plant species and the number of their ITS1 and *trnL* sequences in the insect pollen loads. P values were obtained with Kendall tau rank correlation tests.

As expected from their pollen carrying capacities (see methods), the number of both ITS1 and *trnL* sequences ranked *Hymenoptera *> *Diptera *> *Lepidoptera* (Fig. 3a) with more variability for the ITS1 marker. Among Hymenoptera, *Bombus pascuorum* carried fewer pollen grains and also generated fewer sequences. *Coleoptera* generated surprisingly abundant sequences, especially for ITS1.

We made similar observations for the dominant and mass-flowering species *Rhododendron ferrugineum* (see methods for the choice of this species). The number of *R. ferrugineum* sequences across insect taxa followed the same pattern as that of total sequences (Fig. 3b). Moreover, there was a remarkably significant positive correlation between the mean number of *R. ferrugineum* pollen grains and the mean number of conspecific *trnL* or ITS1 sequences across pollinator groups. Overall, we obtained fewer *R. ferrugineum* sequences with the ITS1 marker as compared to the *trnL,* for *Lepidotera* and *B. pascuorum*.

For both *trnL* and ITS1, we observed a highly positive significant correlation between the number of visits/plant species and the number of sequences of each species in insect pollen loads (all insects pooled, Fig. 3c). Plant species that received the most visits and produced most ITS1 and *trnL* sequences were *R. ferrugineum*, *Hippocrepis comosa, Rubus idaeus, Geranium sylvaticum, Lathyrus linifolius, Genista pilosa, Cardamine pratensis, Potentilla erecta, Ranunculus polyanthemoide* and *Lotus corniculatus.* In contrast, *Rosa pendulina* and *Cerastium arvense* were less visited and produced fewer ITS1 and *trnL* sequences. On the other hand, *Conopodium majus* produced numerous ITS1 sequences even though it received few apparent visits.

## Discussion

In this work, we evaluated the potential of applying DNA metabarcoding to insect pollen loads to describe plant-pollinator interactions. By combining both experimental and field approaches, we demonstrate that metabarcoding is successful at characterizing species composition from both polyfloral pollen mixtures and insect pollen loads. We further show that the method has great potential to indicate quantitative (i.e. using the number of sequences as a direct measure of interactions) or semi-quantitative (i.e. categorizing interactions as weak, medium and strong interaction) plant-pollinator interactions.

### Methodological consideration when applying metabarcoding to pollen samples

It has been advocated that metabarcoding studies should be based on short DNA marker because environmental DNA might be degraded^17^. On the other hand, short markers often lack discriminatory power at fine taxonomic levels. To maximize our ability to identify and quantify the plant composition contributing to insect pollen loads, we used two markers. The P6 loop in *trnL* intron has been shown to be efficient at studying highly degraded DNA in environmental samples^18^, which possibly was the case of the DNA in pollen grains. On the other hand, ITS1 is known to have greater discriminatory power than plastid regions^26^. Both are suitable for identifying most plants at genus or species level^17^,^22^. Among the 402 insect pollen loads studied, 81% and 87% produced *trnL* or ITS1 sequences respectively. Nevertheless, the combined use of both markers resulted in 97% of pollen loads that produced sequences. These results illustrate the advantage of using two markers in such studies^26^.

A critical consideration when working with environmental DNA and sensitive methods such as Illumina sequencing is the problem of contamination and false positives. This risk is especially high in environmental or ancient samples where the DNA is degraded and in very small quantities^17^,^27^,^28^. For the latter, artifactual inclusion of an exogenous contaminant DNA is most likely to generate false positives, as it has the same-if not higher-probability of being amplified than the targeted DNA. This problem might occur for pollen loads as well, in particular when they harbour only a small number of pollen grains. Here, we adopted good laboratory practices by preparing the PCR mixture in a room regularly decontaminated and physically separated from post-PCR work (see Materials and Methods^27^). We further excluded rare sequences from our analysis as recommended by ref. 27. In addition, the plant communities studied here (subalpine ecosystems) were very different from those routinely amplified in our lab (tropical moist forest) or occurring on the campus (plain ecosystem). We believe we obtained such good results not only because of these experimental precautions, but also because the pollen grains were washed from insect bodies prior to DNA extraction. The resulting DNA extraction most likely yielded quantities of DNA from pollen far in excess of any from potential lab contaminants.

### Metabarcoding considerably enlarges the observation window of plant-pollinator interactions

Metabarcoding proved successful in identifying 2.5 more plant species than the sole observation of visits (25 species visited against 63 species identified by metabarcoding) and 92% of species observed to be visited by insects (23 of the 25 species visited). One third of the plant species detected through metabarcoding were recorded in the surrounding areas but not in the communities studied. These are unlikely to result from lab contamination for the reasons explained above. This finding hence suggests that for an even time of prospection, metabarcoding considerably enlarges the spatial and temporal (as pollen may possibly remain on insect bodies for several hours) observation window of plant-pollinator interactions. It has hence the potential to reveal interactions that would have either remained undiscovered or required much more observation time to be detected.

It was noted that 19 entomophilous species known to grow in the communities studied^29^ and for which we had barcodes were not identified in the insect pollen loads. Most of these species were rare on the site or were not at their blooming peak (*Dactylorhiza sambucina*, *Gymnadenia nigra*). Insects hence probably seldom visited these plants, since visitation rates are disproportionately low in sparse populations^30^. Some more abundant undetected plant species were not observed to be visited by insects and possibly were not in insect pollen loads (e.g. *Polygala sp.* personal observations and ref. 31) On the other hand, it is possible that their sequence failed to be properly assigned at the species level and accounted for the large number of sequences assigned at the genus (*e.g. Trifolium*, 99,416 ITS1 and 185,569 *trnL* sequences) or family level (*e.g. Ranunculaceae* 860,874 ITS1 and 12,138 *trnL* sequences).

A final observation was the presence of amphiphilous species (e.g. *Castanea spp*., *Rumex spp*^32^) and anemophilous taxa (Coniferales, Poaceae) in insect pollen loads. Retrieving amphiphilous taxa was less surprising than that of anemophilous ones. This might reflect contact with airborne anemophilous pollens^24^ or active collection of anemophilous pollen when pollen of other species is lacking^32^. A passive process is plausible since grasses are the main component of the herbaceous vegetation matrix and conifers, which are present in the surroundings, produce huge amounts of anemogamous pollen. Whether these passive processes should be included in plant-pollinator networks deserves further investigation.

### The promise and limits of metabarcoding to quantify plant-pollinator interactions

So far, metabarcoding has been successfully used to quantify taxa in airborne pollen^33^ or in honeybee pollen loads^34^ mainly at the genus or family levels. However, another study did not obtain reliable quantitative estimates of the relative abundance of pollen in honeybee pollen loads from metabarcoding data^24^. From mock pollen DNA mixtures we found a systematic positive relationship between the amount of pollen DNA and the number of sequences generated for a given focal species either alone or mixed with other species. The promise of metabarcoding to provide quantitative data is even further supported by the remarkably good consistency, for both markers, between the well-known carrying capacity of insect taxa^29^,^35^,^36^ and the pattern of sequence distribution among these taxa. This relationship was significant despite the noticeably high variability in sequences and pollen abundances (Fig. 3b) within each insect taxon, which might result from the insect behaviour (e.g. grooming activity^37^ or number of plants visited prior to capture). The relationship between insect carrying capacity and number of sequences held true when considering either whole plant communities (all species pooled) or the dominant mass-flowering species *Rhododendron ferrugineum*. For instance, *Hymenoptera* commonly carry proportionally more pollen grains than other insects (especially *Diptera*) and, actually, yielded more sequences. Conversely, *Bombus pascuorum* had smaller pollen loads and also fewer sequences than the other *Bombus* species. In addition, we found that the number of ITS1 and *trnL* sequences obtained for each plant species correlated well with their visit frequency observed in the field. Altogether, these findings suggest that the larger the amounts of pollen on insect bodies, and most likely the greater the number of plant-pollinator interactions the greater the number of sequences yielded.

On the other hand, our experimental approach highlights certain limits that could potentially reduce the reliability of metabarcoding to finely quantify interactions at species level. First, we found a large interspecific variation in extracted DNA yields. Possible causes of this variation may be interspecific differences in pollen wall structure^38^, pollen size^39^,^40^,^41^, genome size^42^, the number of marker copies and DNA extraction efficiency from protoplasts^43^. Therefore, in routine studies these between-species variations will require calibration, for each focal species, of the relationship between the number of pollen grains and the number of sequences. Such calibration will require the best possible estimate of the number of pollen grains extracted. Second, our pollen DNA mixture experiment revealed significant effects of species identity and its interaction with species abundance on the DNA sequences yielded in the DNA mixture, which tended to lose the relationship between the number pollen grain and the amount of DNA sequences yielded. The specificity of the effect suggests that, for certain species, molecules co-extracted with DNA could bias PCR amplification of polyfloral samples. On the other hand, cross-contaminations between the samples we observed could have also contributed to augmenting the variation in the pollen/sequence relationship. Therefore, we believe that these cross-contaminations mostly resulted from the cultivation of the species in the gardening store where they were bought and the manipulation of the flowers in the laboratory. Such experimenter-induced cross-contamination was less likely to occur in our environmental samples. Indeed, insect bodies were collected with a clean net and placed into sterile tubes that were sealed right after collection. A final important point in quantifying plant-pollinator interactions concerns the consistent detection of rare species. Here, our DNA mixture experiments revealed that as few as 5 pollen grains of a target species generated at least 10-fold more sequences than in the negative control (water), supporting the idea that metabarcoding can be successfully used to detect rare pollen, either alone^44^ or in mixtures^33^. However, we observed a large variability in sequence yield principally for the less concentrated DNA mixtures for both markers and the three species. While this variability could be the result of pipetting/sampling bias for small volumes it does not excludes a bias from the metabarcoding process *per se* (DNA extraction, PCR, sequencing). These results question the reliability of the method for assessing weak plant-pollinator interactions. Nevertheless, the least concentrated mixtures corresponded to very few pollen grains of the focal species and, projected to a natural system, to tenuous plant-pollinator interactions.

## Conclusion

Our results suggest that pollen metabarcoding is a much more effective and faster means than pollinator visit observation to detect plant-pollinator interactions in natural communities. Indeed, it revealed interactions that would have needed much more time to be detected or that would have never been observed, especially for plant species found outside the studied communities. Moreover, it will allow the investigation of many pollination networks across large ecological gradients thus opening the opportunity to understand how spatial and biogeographical processes affect pollination interactions and network structure and function. This will help us to better predict the impact of global changes on community stability and biodiversity patterns.

Metabarcoding could also provide invaluable information about individual visitor behaviour and thus resolve networks at an unprecedented level of resolution.

Our results also suggest that metabarcoding has the potential to provide at least semi quantitative data on plant-pollinator interactions, although we are aware that advances should be made in this respect. A more accurate measure of species pollinator efficiency in pollination would require an assessment of their role in conspecific pollen deposition onto plant stigma^45^. Such quantification is an essential step towards the accurate study of plant-pollinator interactions. A fine description of pollinator and stigmatic pollen loads would provide further information not only on plant-pollinator interactions but also on plant-plant interactions. For example, abundant pollen belonging to several plant species in insect pollen loads will indicate that these species frequently share pollinators and interact positively in co-attracting and maintaining the insects in the plant community. In contrast, indirect competition will occur between plant species if they compete for pollinator service or if large quantities of heterospecific pollen are deposited on the stigma. By highlighting these various facets of pollination interactions, metabarcoding will open new avenues of research in network analysis and plant-pollinator interactions and will certainly provide a new perception of pollination networks as a whole.

## Methods

### Artificial pollen DNA mixtures

We used three cultivated species, *Hippeastrum sp.* (HIP, Amaryllidaceae) *Lilium sp.* (LIL, Liliaceae) and *Chrysanthemum sp.* (CHR, Asteraceae), to investigate the intra-and inter-specific variation in the amount of DNA retrieved and to quantify species in pollen mixtures. Our objectives were (1) to investigate if, for a focal species, the number of sequences might be a good proxy for number of pollen grains extracted and (2) if this held true when the pollen of the focal species was mixed with pollen grains of other species with variable abundances. The species were chosen because they are common in most gardening stores (specifically, the store was located in the surroundings of Toulouse about 20 km from the lab) and produce large amounts of pollen that can be easily collected from the stamens.

#### Barcoding plant species used in pollen mixtures

To obtain the reference barcodes for each of the three species, we extracted total DNA from fresh leaves with the DNeasy Plant Mini kit (Qiagen). We then amplified two genomic regions (Table 4): the *trnL* (UAA) intron of the chloroplastic DNA (primers c and d) and the internal transcribed spacer region 1 (ITS1) of the nuclear ribosomal region. These markers are often used as DNA barcodes of plant species^46^,^47^,^48^,^49^,^50^,^51^. The PCR reaction was performed in a total volume of 25 μL containing 1x Greenbuffer (Promega), 200 μM of each dNTP (Promega), 0.4 μM of each forward and reverse primer (Sigma), 1 U of GoTaq (Promega) and less than 10 ng of DNA. For the *trnL* marker, the PCR program was 1 min denaturation at 94 °C, followed by 30 cycles (30 s denaturation at 94 °C, 40 s hybridization at 50 °C, 40 s elongation at 72 °C) with a final elongation at 72 °C for 5 min. The PCR program for ITS1 consisted of 5 min at 90 °C, followed by 30 cycles (30 s denaturation at 94 °C, 40 s hybridization at 54 °C, 1 min elongation 30 s at 68 °C) with a final elongation at 68 °C for 1 min. PCRs were performed in the Thermal Cycler GeneAmp PCR System 9700 (Applied Biosystems). PCR products were visualized on 1% agarose gel and sequenced by Sanger sequencing.

#### Artificial pollen DNA mixture construction

Pollen stock solutions were obtained for each species by vigorously shaking fresh stamens in a 10 mL sterilized tube kept sealed at 4 °C. Three mL of lysis buffer CF solution (Nucleospin Food Kit, Macherey-Nagel) was then added to each tube and thoroughly mixed. The mean number of pollen grains in each stock solution was estimated by counting under a microscope in 10 μl (HIP, LIS) or 2 μl aliquots (CHR, due the higher pollen abundance) with seven replicates per stock solution.

To minimize any loss of extracellular DNA from pollenkitt, pollen solutions were precipitated overnight at −20 °C by adding 2.5 volumes absolute Et-OH and 0.1 volume 3M Na acetate. Tubes were centrifuged at 4,300 g for 30 minutes at 4 °C. Supernatant was then gently removed and the pellets dried under the Microbiological Safety Cabinet (MSC) overnight. Before DNA extraction, 550 μL of lysis buffer (CF; Macherey-Nagel) was added to suspend the pellet.

Total DNA was extracted from 10,000 pollen grains per species with the Nucleospin Food Kit (Macherey-Nagel) in a MSC to avoid contamination. The procedure was repeated five times for each species. DNA concentration was measured in each DNA stock solution using PicoGreen dsDNA Quantitation Reagent; ABI 7900 HT FAST Fluorometer). DNA mixtures were made by adding appropriate volumes of each DNA stock in UHQ H_2_O, as shown in Table 2 with three replicates per mixture. Disrupting pollen walls during DNA extraction by shaking them (30 Hz) with 1 mm glass beads did not improve DNA yields so we eliminated this step from the process.

Sequencing, sequence analysis and plant taxon assignation were performed as explained below. Using the HLfit function of spaMM package^52^ in R software, we tested the effects of pollen DNA amounts of each focal species (*Hippeastrum sp.*, *Chrysanthemum sp.*, *Lilium sp.*), DNA amount of neighbouring species (added to the mixture with the focal species), identity of neighbouring species and interactions between these three factors on the number sequences of the focal species by performing generalized linear model analysis (GLM with a negative binomial error). Starting from the full model, we carried out marginal fitting of terms equivalent to the type II sum of squares in least squares ANOVA.

### Insect pollen loads

#### Visitor and plant sampling

We sampled plants and their visitors in four subalpine *Rhododendron ferrugineum* communities on a 3-km^2^ area in the French Central Pyrenees (southern France) near the village of Camurac (42°46′31″N 01°55′45″E; 1,660 m a.s.l.) during the shrub’s flowering period (June 2012). The site was chosen because both plant and insect communities have previously been studied there^53^,^54^.

In the four communities, entomogamous co-flowering plant species were sampled in order to constitute a reference herbarium of the plant community because some of them were not available in nucleotide databases. To this end, 1 to 2 leaves from at least 2 individuals per species were sampled and transferred into silica gel in the field and then stored at −20 °C before DNA extraction and PCR amplification. New *trnL* and ITS1 barcodes were then obtained for 27 and 58 species respectively as explained above.

In each heathland community, pollinators were captured twice a day (from 10:00 to 11:30 am and from 2:00 to 3:30 pm) during two consecutive days (3h00 total per patch) in a 625 m^2^ area (25 m × 25 m) at the core of each patch. Sampling was conducted using random transect walks. Only insects that contacted fertile parts of the flower were captured. New clean nets were used for each capture and between all sampling sessions to prevent pollen cross contamination between insects. Insects were placed in clean scintillation tubes and stored at 4 °C in the field, then at −20 °C in the lab. Each plant species on which an insect was captured was recorded.

#### Pollen removal from insect bodies

Pollen grains were removed from 402 insect loads by shaking each insect for 10 minutes in either 550 μL or 3 mL lysis buffer (CF: supplied in the Macherey Nagel Food DNA Extraction Kit) for small insects (*e.g.* solitary bees, beetles) or big ones (mainly bumblebees), respectively. Pollen packed in the bee corbiculae (pollen baskets) had been previously discarded as it was no longer involved in pollination process^13^,^55^. The pollen-load solutions were transferred to 15 mL Falcon tubes under a microbiology safety cabinet (MSC) to avoid contamination and pollen DNA was extracted as explained above. The MSC was carefully bleached before and after each extraction.

#### PCR amplification of pollen extracts and sequencing

We used short DNA markers (less than 250 bp) here because the pollenkitt is composed of degraded diploid cells of the parent plant^38^, with possibly degraded DNA, and because of the technical limits of next-generation sequencing. For the chloroplastic marker, we therefore amplified short fragments within the DNA barcodes used for plants (see above), *i.e.* the P6 loop of *trnL* (UAA) intron (primers g and h, Table 4)^48^. We also amplified the ITS1 marker as previously indicated. PCR reactions were performed in 25 μL containing 1x Greenbuffer (Promega), 0.4 μM of each forward and reverse tagged primer (Sigma), 2.5 mM MgCl_2_ heated to 65 °C (Promega), 200 μM of each dNTP (Promega), 1.4 μM BSA (Promega), 1 U AmpliTaq Gold (Fischer) and less than 10 ng template DNA. For the P6 loop of the *trnL* (UAA) intron the PCR program was: denaturation at 94 °C for 1 min; followed by 35 cycles (30 s denaturation at 94 °C, 40 s hybridization at 50 °C, 40 s elongation at 72 °C) and a final elongation 72 °C for 5 min. For the ITS1 marker: 10 min denaturation at 95 °C, followed by 40 cycles (30 s denaturation at 95 °C, 30 s at hybridization 48 °C, 15 s elongation at 72 °C). To retrieve sequences from each sample in bioinformatics analysis, primers were 5′ labelled with a set of 8 bp tags. These were identical on the forward and reverse primers to obtain unique tag combinations for each PCR product. This was done to avoid tag switching events, as recommended in ref. 56. We also performed blank PCR controls. All PCR amplifications were prepared under an UV PCR cabinet to avoid contaminations in a room exclusively devoted to metabarcoding procedures, which is frequently decontaminated and where it is absolutely forbidden to bring/store or handle amplified DNA. Each PCR product was visualized on 1% agarose and quantified using PicoGreen ds DNA Quantitation Reagent. PCRs were mixed depending on these quantities in order to obtain an equimolar pool. The pool was then purified using beads contained in the Illumina TruSeq Nano kit (part #15041758) and libraries were generated from 200 ng of PCR products following the manufacturer’s guide for the Illumina TruSeq Nano kit, except that no sonication was performed. Libraries were sequenced on (1 single run MiSeq Illumina, 2 × 250 pair-end) using the NGS core facility at the Génopole Toulouse Midi-Pyrénées (www.get.genotoul.fr).

### Data analysis

#### Completion of barcode taxonomic reference libraries

We built large barcoding reference libraries by retrieving *trnL* and ITS1 sequences from the EMBL database using the ecoPCR function^57^ (git.metabarcoding.org/obitools/ecopcr/) and enlarged them with our own sequences (see above). To focus the assignments on the species or genera (see below) of the studied site, we derived from the previous EMBL libraries (EMBL_databases), two smaller reference libraries containing only the sequences of either the entomogamous species (local_species_database) or genera (local_genus_database) growing at the study site. We then used obiuniq from the OBITools program^58^ to dereplicate the sequences. When identical barcodes were obtained for different species, the sequence was assigned to the last common ancestral taxon. Overall, the local reference libraries contained all the genera and 54 of the 66 (82%) entomogamous species occurring in the four patches studied.

#### Sequence analysis and plant taxon assignation

Paired-end reads were first assembled using FLASH^59^ to be merged into extended single sequences, by taking into account the read overlapping quality. Next, sequences were demultiplexed using the ngsfilter command of the OBITools^58^ package by allowing 0 errors on tags and a maximum of two errors on primers. Finally, sequences containing ambiguous nucleotides shorter than 50 bp for the ITS1 marker, were discarded. The remaining sequences were finally dereplicated using the obiuniq command included in the OBITools package. This analysis of more than 140 million reads was performed on the bioinfo.genotoul.fr cluster. For the sequences from pollen DNA mixtures, a unique taxon was assigned to each unique sequence using the ecoTag program based on a global alignment algorithm^60^ and the EMBL_database (see above). Because our mixtures were built with pollen DNA from three species belonging to different families, the final assignment of each sequence was done at the family level. For the sequences from the insect pollen loads, we applied two additional cleaning steps. First, we removed all sequences for which the number of counts was below 1 per thousand of the most common sequences and the sequences for which the maximum number of counts per sample was lower than 10. Then, a unique taxon was assigned to each unique sequence using the ecoTag program that successively compared our sequences to the three taxonomic reference libraries: the local_species_database, the local_genus_database and lastly the EMBL_database (see above). At each step, a taxonomic assignation was retained if it was at least at the genus level and with a best match score (i.e. % identity) above 95 or 98%. For each marker and reference library, the best match score threshold value was chosen by observing modes in its distribution.

#### Quantifying interaction in plant-pollinator communities

We assessed whether metabarcoding may provide any help in quantifying plant-pollinator interactions by examining: (1) How the total amount of sequences generated from the pollen loads varies among insect taxa. From a previous study based on 522 visitors in 17 *R. ferrugineum* heathlands distributed across the Pyrenees^35^ we have a good picture of the carrying capacity of insect taxa. The average number of pollen grains in individual pollen loads can be ranked: *Hymenoptera* (*Apis mellifera *≈ *Bombus lucorum *≈ other wild bees; from 2100 to 2500 pollen grains > *Bombus pascuorum* (≈800 grains) > *Diptera* (*Syrphidae *≈ 500 grains > *Empididae *≈ 80 grains) and *Lepidoptera* (≈20 grains). The few recorded *Coleoptera* carried up to 200 grains. If the number of sequences mirrors the number of pollen grains then, the number of ITS1 and *trnL* sequences would scale across insect taxa in the same order as their carrying capacities; (2) If, when considering a target plant species (here *R. ferrugineum*), there is a relationship between the number of pollen grains in the pollen loads and the number of conspecific ITS1 and *trnL* sequences. Overall, we would expect a quite similar trend as for total pollen analysis even if the proportion of *R. ferrugineum* in pollen loads is usually lower in *Diptera* (0.29–0.55) than in *Hymenoptera* (0.61–0.89; but see *Bombus pascuorum:* 0.41^35^. Honeybees and bumblebees are indeed usually reported as the main *R. ferrugineum* pollen carriers in *R. ferrugineum* heathlands in the Pyrenees^29^ and in the Alps^36^, while *Diptera* usually display much smaller *R. ferrugineum* pollen loads; (3) If there is a positive correlation between the number of visits (all visitors pooled) received by a plant species and the number of its ITS1 and *trnL* sequences. For (2) and (3), correlation significance was assessed with the Kendall’s rank correlation test.

## Additional Information

**How to cite this article**: Pornon, A. *et al.* Using metabarcoding to reveal and quantify plant-pollinator interactions. *Sci. Rep.* **6**, 27282; doi: 10.1038/srep27282 (2016).

## Supplementary Material

Supplementary Information

## Figures and Tables

**Table 1:** Analysis of the number of sequences obtained from experimental DNA mixtures.

Focal species	*Hippeastrum sp.*		*Chrysanthemum sp.*		*Lilium sp.*	
Source of variation	ITS1	*trnL*	ITS1	*trnL*	ITS1	*trnL*
DNA focal sp.	***	***	***	***	***	***
Neighbour identity (id.)	***	***	***	***	***	***
Neighbour DNA amount	**	ns	ns	ns	ns	ns
DNA focal sp. X neighbour id.	**	ns	ns	ns	ns	ns
DNA focal sp. X neighbour DNA amount	ns	ns	ns	ns	ns	ns
Neighbor id. X neighbour DNA amount	***	ns	***	ns	*	**
DNA focal sp. X neighbour id. X neighbour DNA amount	ns	ns	ns	ns	ns	ns

A negative binomial model was fitted to the data with DNA amounts of the focal species, the identity and DNA amount of added species (neighbours) in the mixtures as explanatory variables. Starting from the full model, we carried out marginal fitting of terms equivalent to the type II sum of squares in least squares ANOVA (see supplementary Table S1 for all data of the negative binomial model).

^***^P < 0.001; ^**^P < 0.01; ^*^P < 0.05; ns, not significant.

**Table 2:** Pollen DNA mixtures (with corresponding pollen numbers in brackets) performed to test the effect of pollen DNA amounts and mixtures on the number of sequences yielded for the 3 focal species.

	DNA (ng)	*Hippeastrum sp.*				*Lilium sp.*			
		0 (0)	0.25 (5)	2.5 (50)	7.5 (150)	0 (0)	0.2 (4)	2 (40)	4 (80)
*Lilium sp.*	0	*	*	*	*				
	0.2	*	*	*	*				
	2	*	*	*	*				
	4	*	*	*	*				
*Chrysanthemum sp.*	0	*	*	*	*	*	*	*	*
	0.025 (125)	*	*	*	*	*	*	*	*
	0.05 (350)	*	*	*	*	*	*	*	*
	0.25 (1750)	*	*	*	*	*	*	*	*

**Table 3:** Plant taxa present on the study site, identified or not detected by metabarcoding (MBC) in the insect pollen loads and number of ITS1 and *trnL* sequences for each taxon.

	ITS1 seq.	*trnL seq.*		ITS1 seq.	*trnL seq.*		ITS1 seq.	*trnL seq.*
**Taxa present in the studied patches, detected by MBC**								
Ajuga reptans*	0	146,220	Thalictrum aquilegiifolium*	0	1,449,412			
Alchemilla vulgaris*	0	2,873	*Thesium alpinum*	112,784	1,346	*Cytisus scoparius*	0	3,128,930
Anthyllis vulneraria*	0	55,887	*Thymus serpyllum*	13,544	8,484	*Epilobium montanum*	0	8,722
Biscutella laevigata*	11,872	0	*Trifolium repens*	0	2,102	*Filipendula ulmaria*	0	3,609
Bistorta officinalis*	0	575,118	*Trifolium*	99,416	185,569	*Gentiana*	0	18,141
Cardamine pratensis*	1,157,851	1,396,787	*Trollius europaeus*	0	691,893	*Helianthemum nummularium*	0	256,788
Cerastium arvense*	8,312	31,424	*Valeriana pyrenaica*	82,809	0	*Lonicera*	0	17,884
*Chamaespartium sagitalis*	0	977,464	Veronica chamaedrys*	0	528,218	*Lonicera nigra*	0	4,245
Conopodium majus*	6,814,400	286,257	**Taxa present in the studied patches, not detected by MBC**			*Medicago sativa*	0	4,570
*Cruciata glabra*	19,714	15,226	*Adenostyles alliariae*	0	0	*Pimpinella major*	0	29,166
*Galium*	0	75,490	*Arabis alpina*	0	0	*Phyteuma orbiculare*	0	314,037
Genista pilosa*	5,345,674	769,886	*Dactylorhiza sambucina*	0	0	*Pseudorchis albida*	0	35,544
Geranium sylvaticum*	2,704,313	1,812,125	*Doronicum austriacum*	0	0	*Silene dioica*	0	34,304
*Geum rivale*	18,582	14,900	*Doronicum pardalianches*	0	0	*Tilia*	0	98,322
Hieracium hoppeanum*	58,020	0	*Globularia nudicaulis*	0	0	*Vaccinium myrtillus*	0	32,449
*Hieracium laevigatum*	11,636	0	*Gymnadenia nigra*	0	0	*Valeriana officinalis*	0	28,863
Hippocrepis comosa*	10,181,447	1,571,634	Melampyrum sylvaticum*	0	0	*Veratrum album*	0	12,595
Lathyrus linifolius*	1,454,712	1,264,673	*Paris quadrifolia*	0	0	*Vicia*	0	6,972
*Lathyrus pratensis*	0	25,067	*Polygala calcarea*	0	0	**Anemophilous/amphiphilous taxa detected by MBC**		
*Leontodon hispidus*	189,110	424,733	*Polygala vulgaris*	0	0	*Anthoxanthum alpinum*	20,629	0
Lotus corniculatus*	9,347,981	2,607,881	*Ranunculus carinthiacus*	0	0	*Abies*	0	75,215
Melampyrum pratense*	22,054	25,520	*Saxifraga granulata*	0	0	*Agrostis gigantea x Agrostis stolonifera*	23,197	0
*Myosotis arvensis*	0	135,170	*Saxifraga paniculata*	0	0	*Castanea*	0	304,284
*Narcissus*	0	111,249	*Trifolium alpinum*	0	0	*Coniferales*	0	1,781,966
*Orchidaceae*	121,245	0	Trifolium pratense*	0	0	*Cupressaceae*	0	97,527
*Pedicularis foliosa*	133,710	54,351	*Viola canina*	0	0	*Dactylis glomerata*	0	19,013
Potentilla erecta*	1,070,704	1,257,196	*Viola cornuta*	0	0	*Fagaceae*	0	67,555
*Ranunculus aconitifolius*	612,552	93,209	*Viola riviniana*	0	0	*Fagus*	0	9,087
*Ranunculus acris*	0	73,471	**Taxa present in the surrounding landscape, detected by MBC**			*Luzula nivea*	0	4082
Ranunculus polyanthemoides*	4,434,795	1,239,768	*Achillea millefolium*	0	18,361	*Plantago*	73,539	64,609
*Rhinanthus alectorolophus*	188,868	0	*Allium cepa*	71,640	0	*Poa*	38,8799	8,371
Rhododendron ferrugineum*	13,571,521	6,210,561	*Angelica sylvestris*	0	7,498	*Poa alpina*	76,922	0
Rosa pendulina*	255,573	218,044	*Arnica*	0	15,439	*Poa chaixii*	789,414	0
Rubus idaeus*	2,596,786	638,259	*Bellis perennis*	0	7,065	*Poaceae*	375,188	2,107,304
*Sanguisorba minor*	0	17,771	*Capsella rubella*	0	13,814	*Quercus*	0	8,204
*Senecio*	0	84,653	*Chaerophyllum hirsutum*	2,614,364	56,496	*Rumex acetosa*	0	6,001
*Stellaria*	0	10,636	*Convolvulus arvensis*	213,219	0			
Taraxacum officinale*	220,907	0	*Crepis mollis*	32,581	0			

*Taxa visited during the capture sessions.

**Table 4:** List of primers used in this study.

DNA region	Primer name	Primer sequence 5′-3′	Reference	Amplicon average length(bp)
*trnL* (UAA)	c	CGAAATCGGTAGACGCTACG	Taberlet *et al.*^46^	569
	d	GGGGATAGAGGGACTTGAAC	Taberlet *et al.*^48^	51
*trnL* (UAA) P6 loop	g	GGGCAATCCTGAGCCAA	Baamrane *et al.*^50^	280
	h	CCATTGAGTCTCTGCACCTATC	White *et al.*^51^	
ITS1	ITS1-F	GATATCCGTTGCCGAGAGTC		
ITS1	ITS1-R	GGAAGTAAAAGTCGTAACAAGG		

Amplicon length is reported on the basis of sequences obtained in this study.
