# Supplementary material for: Using metabarcoding to reveal and quantify plant-pollinator interactions
Source: Sci Rep. 2016 Jun 3;6:27282. doi: 10.1038/srep27282 (PMC4891682; doi:10.1038/srep27282)
Supplement: Supplementary Information [file srep27282-s1.pdf]

Using metabarcoding to reveal and quantify plant-pollinator interactions

André Pornon[1,2]*‡, Nathalie Escaravage[1,2]*, Monique Burrus[1,2], Hélène Holota[1,2], Aurélie Khimoun[3], Jérome Mariette[4], Charlène Pellizzari[1,2], Amaia Iribar[1,2], Roselyne Etienne[1,2], Pierre Taberlet[5], Marie Vidal[6], Peter Winterton[7], Lucie Zinger[1,2], Christophe Andalo[1,2]*.

[1]Laboratoire Evolution and Diversité Biologique EDB, Université Toulouse III Paul Sabatier, F-31062 Toulouse, France,

[2]CNRS, EDB, UMR 5174, F-31062 Toulouse, France

andre.pornon@univ-tlse3.fr; nathalie.escaravage@univ-tlse3.fr; monique.burrus@univ-tlse3.fr; helene.holota@univ-tlse3.fr; charlene.pellizzari@live.fr; amaya.pelozuelo@univ-tlse3.fr; roselyne.etienne@univ-tlse3.fr; lucie@zinger.fr, christophe.andalo@univ-tlse3.fr;

[3] Laboratoire Biogeosciences, Université de Bourgogne 6 bld Gabriel, F-21000 Dijon, France. aurelie.khimoun@gmail.com

[4] Plate-forme Bio-informatique Genotoul, Mathmatiques et Informatique Appliques INRA, UR875 Toulouse, F-31320 Castanet-Tolosan, France. jerome.mariette@toulouse.inra.fr

[5]Laboratoire d'Ecologie Alpine, CNRS UMR 5553, Université Joseph Fourier, BP 43, F-38041 Grenoble, France. pierre.taberlet@ujf-grenoble.fr

[6]GeT-PlaGe, Genotoul, INRA UAR1209, F-31320 Castanet-Tolosan, France. marie.vidal@toulouse.inra.fr

[7]Département de Langues et Gestion, Université Paul Sabatier, F-31062 Toulouse, France. peter.winterton@univ-tlse3.fr

‡ Correspondence: andre.pornon@univ-tlse3.fr

Supplementary information

Table S1. Data of the negative binomial model of sequences number analyses of *Hippeastrum sp.*, *Chrysanthemum sp.* or *Lilium sp.* obtained from experimental DNA mixtures. DNA amounts of the focal species, the identity and DNA amount of added species (neighbours) in the mixtures are explanatory variables. Starting from the full model, we carried out marginal fitting of terms equivalent to the type II sum of squares in least squares ANOVA.

Focal species : *Hippeastrum sp.*	ITS1			*trnL*		
Source of variation	*Likelihood ratio*	*df*	*P-value*	*Likelihood ratio*	*df*	*P-value*
DNA focal sp.	73.460	1	<0.0001	21.742	1	<0.0001
Neighbour identity (id.)	62.2864	3	<0.0001	52.864	3	<0.0001
Neighbour DNA amount	7.3944	1	0.0065	0.1598	1	0.6894
DNA focal sp. X neighbuor id.	13.122	2	0.0014	3.126	2	0.2095
DNA focal sp. X neighbour DNA amount	2.011	1	0.1562	0.042	1	0.8376
Neighbor id. X neighbour DNA amount	14.925	2	0.0005	0.585	2	0.7465
DNA focal sp. X neighbour id. X neighbour DNA amount	0.895	1	0.3440	1.050	1	0.3055

Focal species : *Chrysanthemum sp.*	ITS1			*trnL*		
Source of variation	*Likelihood ratio*	*df*	*P-value*	*Likelihood ratio*	*df*	*P-value*
DNA focal sp.	13.6939	1	0.0002	18.040	1	<0.0001
Neighbour identity (id.)	43.052	3	<0.0001	31.976	3	<0.0001
Neighbour DNA amount	1.4775	1	0.2242	2.236	1	0.1353
DNA focal sp. X neighbour id.	0.0013	2	0.9993	0.056	2	0.9725
DNA focal sp. X neighbour DNA amount	0.599	1	0.4390	0.287	1	0.5922
Neighbor id. X neighbour DNA amount	17.142	2	0.0002	3.325	2	0.1896
DNA focal sp. X neighbour id. X neighbour DNA amount	0.562	1	0.4533	0.057	1	0.8110

Focal species : *Lilium sp.*	*ITS1*			*trnL*		
Source of variation	*Likelihood ratio*	*df*	*P-value*	*Likelihood ratio*	*df*	*P-value*
DNA focal sp.	32.305	1	<0.0001	36.819	1	<0.0001
Neighbour identity (id.)	60.842	3	<0.0001	30.669	3	<0.0001
Neighbour DNA amount	0.006	1	0.9364	0.4110	1	0.5214
DNA focal sp. X neighbour id.	3.312	2	0.1909	3.1059	2	0.2116
DNA focal sp. X neighbour DNA amount	0.5681	1	0.4510	1.2853	1	0.2569
Neighbour id. X neighbour DNA amount	6.600	2	0.0369	13.010	2	0.0015
DNA focal sp. X neighbour id. X neighbour DNA amount	1.205	1	0.2723	0.9845	1	0.3211

Table S2. Insects captured in plant communities and analyzed for ITS1 and *trnL* sequences of plant species in their pollen loads.

			Number of individuals	
		**Insect species**	**with sequences**	**without sequences**
**Hymenoptera**	Apidae	*Apis mellifera*	35	0
	Apidae	*Bombus lucorum*	33	0
	Apidae	*Bombus pascuorum*	13	1
	Apidae	*Bombus pratorum* *	5	0
		**Other Bombus**		0
	Apidae	*Bombus wurflenii* ‡	36	2
	Apidae	*Bombus gerstaeckeri* *	1	0
	Apidae	*Bombus lapidarius* *	2	1
	Apidae	*Bombus ruderarius**	1	0
	Apidae	*Bombus soroeensis* *	1	0
	Apidae	*Bombus sylvestris*	0	1
	Apidae	Unidentified	6	0
		**Wild bees ‡**		
	Andrenidae	*Andrena sp*	3	0
	Halictidae	*Halictus sp*	1	0
	Halictidae	*Lasioglossum albipes*	11	**0**
	Halictidae	*Lasioglossum calceatum*	1	0
	Halictidae	*Lasioglossum fratellum*	1	0
	Unidentified	Unidentified	9	0
		**Other Hymenoptera**		
	Tenthredinidae	*Tenthredo mesomela* †	1	0
	Tenthredinidae	Unidentified	2	0
**Diptera**	Empididae	*Empis empis sp*	1	0
	Empididae	*Empis euempis ciliata*	6	0
	Empididae	*Empis euempis tessellata*	24	0
	Empididae	*Empis leptempis pandellei*	53	5
	Empididae	*Empis sp*	1	0
	Empididae	*Empis xanthempis testacea*	1	0
	Empididae	Unidentified	4	0
	Syrphidae	*Cheilosia albitarsis ranunculi*	1	0
	Syrphidae	*Cheilosia nigripes*	1	0
	Syrphidae	*Eristalis jugorum*	1	0
	Syrphidae	*Eristalis nemorum*	2	0
	Syrphidae	*Eumerus sp*	1	0
	Syrphidae	*Lapposyrphus lapponicus*	2	0
	Syrphidae	*Melanostoma dubium*	2	0
	Syrphidae	*Melanostoma melaria*	1	0
	Syrphidae	*Melanostoma mellinum*	3	0
	Syrphidae	*Parasyrphus vittiger*	1	0
	Syrphidae	*Platycheirus albimanus*	1	0
	Syrphidae	*Platycheirus melanopsis*	1	0

	Syrphidae	*Platycheirus tarsalis*	1	0
	Syrphidae	*Rhingia campestris*	1	0
	Syrphidae	*Sphaerophoria batava*	13	0
	Syrphidae	*Sphaerophoria infuscata*	15	1
	Syrphidae	*Sphaerophoria interrupta*	4	0
	Syrphidae	*Sphaerophoria scripta*	7	0
	Syrphidae	*Syrphus ribesii*	2	0
	Syrphidae	*Volucella bombylans*	18	0
	Syrphidae	Unidentified	1	0
	Muscidae	*Helina reversio* *	1	0
	Muscidae	*Thricops nigritellus* *	2	0
	Calliphoridae	*Bellardia pandia* †	1	0
	Bombyliidae	*Bombylius major* †	1	0
	Anthomyiidae	*Chiastocheta rotundiventris* †	1	0
	Anthomyiidae	*Hylemya vagans* †	1	0
	Agromyzidae	*Melanagromyza sp* †	1	0
	Anthomyiidae	*Pegoplata aestiva* †	2	0
	Sciomyzidae	*Pherbellia cinerella* †	1	0
	Sarcophagidae	*Sarcophaga subvicina* †	1	0
**Coleoptera**	Cantharidae	*Cantharis rustica* ‡	1	0
	Chrysomelidae	*Cryptocephalus aureolus* ‡	2	0
	Chrysomelidae	*Cryptocephalus sericeus* ‡	2	0
	Elateridae	*Ctenicera cuprea*	0	1
	Melyridae	*Dasytes niger* ‡	1	0
	Oedemeridae	*Oedemera virescens* ‡	12	0
**Lepidoptera**	Nymphalidae	*Aglais urticae*	0	1
	Lycaenidae	*Callophrys rubi*	1	0
	Nymphalidae	*Clossiana euprhosyne*	1	0
	Nymphalidae	*Coenonympha pamphilus*	4	0
	Lycaenidae	*Cupido minimus*	3	0
	Geometridae	*Epirrhoe tristata*	2	0
	Nymphalidae	*Erebia oeme*	1	0
	Hesperiidae	*Erynnis tages*	1	0
	Noctuidae	*Euclydia glyphica*	4	0
	Nymphalidae	*Euphydryas aurinia*	3	0
	Geometridae	*Isturgia sp*	1	0
	Nymphalidae	*Melitaea diamina*	1	0
	Nymphalidae	*Melitaea sp*	2	0
	Pieridae	*Pieris napi*	2	0
	Unidentified	Unidentified	2	0
**Total insects**			389	13

Because some insect species or species groups had a small number of individuals, were phylogenetic unrelated or/and had very different morphologies they were not considered in certain analyses of the Fig 3

* Insect species or group of species not considered in Fig 3a

‡ Insect species or group of species not considered in Fig 3b

† Insect species or group of species not considered in both Fig 3b and Fig 3a
